# Radiation induced vaporization of exoskeletal droplets as potential x‐ray acoustic contrast agents

**DOI:** 10.1002/mp.18017

**Published:** 2025-08-08

**Authors:** Mohamed Elsayed Eldib, William N. Frantz, Mark A. Borden, Ryan Lanning, Moyed Miften, David H. Thomas

**Affiliations:** ^1^ Department of Radiation Oncology University of Colorado School of Medicine Aurora Colorado USA; ^2^ Biomedical Engineering Program University of Colorado Boulder Colorado USA; ^3^ Department of Mechanical Engineering University of Colorado Boulder Colorado USA; ^4^ Department of Radiation Oncology Thomas Jefferson University Philadelphia Pennsylvania USA

## Abstract

**Background:**

In vivo dosimetry is a crucial component of ensuring accurate and safe radiation therapy (RT) delivery, but many existing techniques face challenges such as low signal‐to‐noise ratio (SNR), which can limit their clinical applicability.

**Purpose:**

In this study, we investigate a novel contrast agent sensitive to megavoltage (MV) radiation in vitro, ultimately aiming for in vivo dosimetry.

**Methods:**

Vaporizable exoskeletal droplets were engineered to phase‐change into ultrasound‐responsive microbubbles upon exposure to MV photon radiation. These droplets comprised a hydrocarbon (HC) exoskeleton doped with gold nanoparticles (GNPs) surrounding a liquid fluorocarbon (FC) core. Radiation absorbed by the GNPs induced localized heating, leading to vaporization of the FC phase. Droplet vaporization in response to clinical MV radiation was observed under a microscope at varying temperatures. Individual droplet samples were heated to temperatures ranging from 31 to 34°C, incubated for 8 min, then irradiated with a 5 × 5‐cm^2^ 10 × flattening filter free (FFF) photon beam (Varian Truebeam) at a dose rate of 24 Gy/min to measure radiation‐induced vaporization. *T*‐tests (α = 0.05) were performed comparing the number of bubbles generated from irradiated droplets with GNPs compared to irradiated droplets without GNPs and nonirradiated droplets with GNPs.

**Results:**

GNP‐doped exoskeletal droplets exhibited enhanced vaporization in response to MV radiation compared to heating alone. Vaporization increased with radiation dose, and the dose threshold required for vaporization decreased with rising temperature. Specifically, at 31 and 34°C, the dose required to vaporize 10% of bubbles (D10%) decreased from 22 to 4 Gy, and that required to vaporize 50% of bubbles (D50%) decreased from 67.5 to 36 Gy, respectively. The activation threshold at body temperature (37°C) was extrapolated to be clinically relevant, with D10% activation estimated to be 0.41 Gy. At *T* = 32 to 34°C, we showed statistically significant radiation‐induced vaporization of droplets with GNPs compared to non‐irradiated droplets with GNPs (*p*‐values from 0.0003 to 0.0232). Irradiation of droplets lacking GNPs did not induce notable vaporization.

**Conclusions:**

GNP‐doped exoskeletal droplets were demonstrated to exhibit enhanced vaporization upon exposure to clinically relevant MV x‐ray radiation doses compared to thermal activation alone in the absence of radiation. This is the first step in the development of an x‐ray acoustic contrast agent for dosimetry of RT in vivo.

## 1 INTRODUCTION

Radiation dosimetry (measuring the amount and distribution of radiation a patient receives) is a crucial aspect of radiation therapy (RT) to ensure that the correct radiation dose is accurately delivered, allowing for better tumor control and lower side effects from off‐target radiation to surrounding healthy tissues. In vivo dosimetry tools play a crucial role in measuring patient radiation exposure during treatment. Existing in vivo dosimetry tools, however, are limited in providing a comprehensive three‐dimensional (3D) dose distribution. Thermoluminescent detectors (TLDs) offer only point measurements on the skin,^1^ while GafChromic films are confined to two‐dimensional (2D) assessments on the surface of a phantom or patient. Although electronic portal imaging devices (EPIDs) have been explored as 3D patient‐specific dosimetry tools,^2^, ^3^ calibration uncertainties and dose reconstruction challenges persist, hindering their widespread adoption.^4^, ^5^

The x‐ray acoustic (XA) effect has been proposed as a method for real‐time 3D in vivo dosimetry, but this method has not yet been realized clinically due to deficiencies in both signal‐to‐noise ratio (SNR) and reconstruction algorithms. The XA effect follows the same principles as the photoacoustic effect:^6^ acoustic waves are induced following the absorption of heat energy by the tissue from a pulsed photon or proton beam. The XA effect occurs when tissue absorbs the high‐energy pulsed x‐ray radiation, and the XA signal generated by clinical LINACs during RT has been shown to be proportional to the absorbed radiation dose.^7^, ^8^ By detecting the acoustic waves using ultrasound, an image of the entire dose distribution within a patient can be reconstructed.^9^ Unlike conventional diagnostic acoustic imaging techniques that image the structure of tissue, the aim of XA dosimetry is to image the acoustic sources induced by the x‐ray dose deposition within the patient. However, a drawback of the x‐ray acoustic effect lies in its intrinsically low SNR, thereby limiting its clinical utility for in vivo dosimetry.^10^, ^11^

Gas‐filled lipid‐coated microbubbles are commonly used as ultrasound contrast agents for echocardiography,^12^ super‐resolution ultrasound localization microscopy,^13^ and ultrasound molecular imaging.^14^ Commercial microbubble ultrasound contrast agents have been tested for sensitivity to MV x‐ray radiation, but they demonstrated variability between agents and limited utility due to the relatively small change in acoustic properties (3.5‐dB reduction for the maximum dose).^15^ Phase‐change droplets, on the other hand, have yielded more promising results as potential x‐ray acoustic contrast agents. Phase‐change droplets comprise a vaporizable liquid core that can generate an echogenic microbubble upon exposure to an energy stimulus (thermal, acoustic, optical, x‐ray etc.).^16^, ^17^, ^18^, ^19^ The vaporization event itself can enhance the photoacoustic signal,^20^ or the echogenic microbubble can be imaged subsequently with pulse‐echo ultrasound.^21^ When heated to a metastable state above their boiling point (superheated), phase‐change droplets have been shown to be radiosensitive to both proton and photon energy sources.^21^, ^22^, ^23^, ^24^, ^25^, ^26^, ^27^, ^28^

For photon‐induced vaporization, perfluorobutane (C_4_F_10_, *b.p*. = −2.1°C) polymer‐coated nanodroplets were investigated as potential in vivo dosimeters due to their relatively high degree of superheat near physiological temperature.^26^, ^29^ Carlier et al. showed little vaporization at physiological temperature in response to 6 MV clinical photon beam, with slightly stronger vaporization with energy of 15 MV.^26^ Upon heating the droplets to 65°C and using a 6 MV beam, a strong vaporization response was observed,^26^ consistent with previous observations of the same droplet type.^29^ The sensitization of the droplets at 65°C is supported by the theoretical levels of superheat required for photon sensitization predicted by d'Errico et al.^27^ As proposed by the group, a potential approach to achieving vaporization at a more clinically relevant radiation dose and temperature could involve utilizing a perfluoropropane (C_3_F_8_, *b.p*. = −36.7°C) or heptafluoropropane (C_3_HF_7_, *b.p*. = −18°C) core. However, the superheat of perfluoropropane would be near its predicted thermodynamic limit (spinodal),^27^, ^28^ leading to spontaneous vaporization at body temperature. Lower boiling point compounds (i.e., C_3_F_8_, C_3_HF_7_, C_4_F_10_) also suffer from decreased colloidal stability and increased fugacity,^17^, ^30^, ^31^ where fugacity describes the escaping tendency of the fluorocarbon (FC) and is equivalent to the partial pressure. Increased fugacity can lead to dissolution of the droplets and mass transfer from the droplets to microbubbles, causing bubble inflation and safety concerns.^17^, ^31^ Droplets made with higher boiling point FCs (i.e., C_5_F_12_, C_6_F_14_) with lower fugacity are resistant to dissolution and bubble inflation^31^ but require higher energies for vaporization.^17^, ^30^ A further discussion on the colloidal stability of FC droplets is provided in the Supplementary Material.

Shakya et al. designed multi‐phase droplets as a means to lower the vaporization energy of higher boiling point FCs, such as C_5_F_12_.^32^, ^33^ These droplets consist of a liquid C_5_F_12_ phase and a solid hydrocarbon (HC) phase. Depending on the surfactant utilized, the droplets will take on an HC‐in‐FC (endoskeletal) or an FC‐in‐HC (exoskeletal) morphology. Localized melting of the HC phase as the HC/FC interface induces mixing with the FC phase. This interfacial mixing disrupts the intermolecular cohesion of the FC phase and depresses the spinodal, leading to vaporization. By selection of an appropriate HC, the vaporization temperature of the droplet can be tuned to the desired vaporization energy. Such endoskeletal droplets were recently developed as photoacoustic contrast agents.^34^ By incorporating a near‐infrared absorbing dye into the HC phase, Silwal et al. demonstrated enhanced photothermal vaporization of the endoskeletal droplets when compared to liquid FC droplets without a solid HC phase. Although more work has been done investigating the endoskeletal droplets, exoskeletal droplets are expected to have better biocompatibility; exoskeletal droplets are stabilized by lipid, whereas the endoskeletal droplets are stabilized by a potentially non‐biocompatible fluorosurfactant.

Prior work in designing phase‐change droplets as photoacoustic contrast agents have incorporated gold nanoparticles (GNPs), due to their photothermal properties.^20^, ^35^ Upon laser irradiation of these droplets, absorption of the light causes localized heating around the GNPs, leading to vaporization of the droplet. Nanoscale effects of MV photon radiation interaction with GNPs in aqueous media have been modelled by Monte Carlo simulations and predict highly inhomogeneous dose distributions in the local microenvironment. Low‐energy Auger electrons with a range of < 200 nm have been shown to produce up to 1000 × dose enhancement in the immediate vicinity of GNPs (< 1µm).^36^, ^37^ Inspired by these results, we designed exoskeletal droplets doped with GNPs and investigated their vaporization in response to MV x‐ray radiation. Our hypothesis posits that the dose enhancement resulting from GNP photon absorption induces localized heating and mixing at the HC/FC interface, leading to vaporization. Furthermore, we propose that the resulting microbubbles can serve as contrast agents, detectable via ultrasound imaging, for x‐ray acoustic in vivo dosimetry during radiation therapy (RT). This approach holds promise for real‐time in vivo dose verification in the context of adaptive radiotherapy (ART) and FLASH‐RT applications.

## 2 METHODS

### 2.1 Materials

The following chemicals were used as received: n‐perfluoropentane (C_5_F_12_, 99%, FluoroMed, Round Rock, TX, USA) and we refer to it as FC; 1,2‐dibehenoyl‐sn‐glycero‐3‐phosphocholine (DBPC) (>99%, Avanti Polar Lipids, Alabaster, AL, USA); 1,2‐disteroly‐sn‐gylcero‐3‐phosphoethanolamine‐N‐[methoxy(polyethylene glycol)‐5000] (DSPE‐PEG5K) (>99%, Avanti Polar Lipids, Alabaster, AL, USA); n‐eicosane (*n*‐C_20_H_42_, 99%, Beantown Chemical, Hudson, NH, USA) and we refer to it as C20; n‐heneicosane (*n*‐C_21_H_44_, >99%, TCI, Portland, OR, USA) and we refer to it as C21; chloroform (99.8%, Fisher Scientific, Waltham, MA, USA); and 10x phosphate buffered saline (PBS) (Molecular Biologicals International, Irvine, CA, USA), filtered. FITC‐labeled organic spherical GNPs in chloroform, 1.8nm in diameter (E11‐1.8‐FITC‐CHL‐2.5) were obtained from Nanopartz Inc. (Loveland, CO, USA).

#### 2.1.1 Fabrication of exoskeletal droplets with GNPs

A lipid suspension was first prepared with DBPC and DSPE‐PEG5K (9:1 molar ratio) dissolved in chloroform in a 20 mL glass vial. Although stirring, the chloroform was removed by flowing N_2_ gas over the headspace until most of the liquid was removed (∼ 10–20 min). The resulting film was dried under vacuum overnight. The dried lipid film was rehydrated in PBS, then sonicated with a tip sonicator (Branson Sonifier 450) for 10 min at low power (3/10) to convert multilamellar vesicles to unilamellar liposomes. This lipid suspension was then used to generate exoskeletal droplets as described below.

**FIGURE 1:**
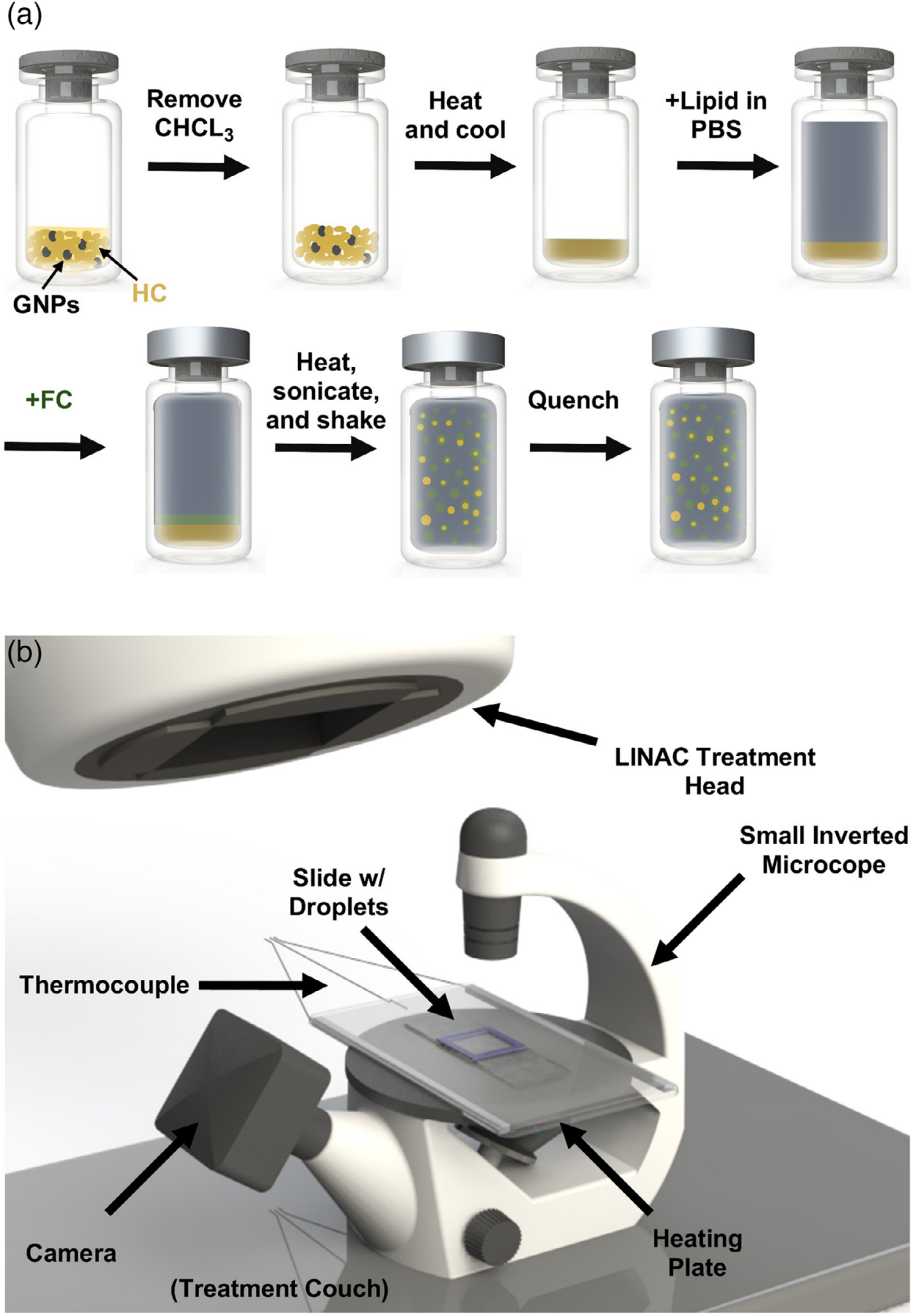
(a) Schematic of the synthesis of exoskeletal droplets with GNPs. (b) Schematic of the MV radiation beam‐based droplet vaporization setup. GNP, gold nanoparticles; MV, megavoltage.

The general approach for producing exoskeletal droplets with GNPs is shown in Figure 1a. The approach was derived from a shaking technique described in Shakya et al. 2020.^33^ The solid HC was weighed (60 mg) in a 3 mL glass vial, then dissolved in chloroform. The HC was transferred to a different 3 mL vial containing approximately 0.6 mg of GNPs (1% w/w) in chloroform. The chloroform was removed, and the HC and GNPs were dried overnight. No stirring was used during removal of the solvent to avoid loss of GNPs due to deposition onto the stir bar. The HC and GNPs were then heated in a hot bath to 5°C above the HC melting temperature (C20 or C21), then quenched to recrystallize the HC, forming a film. Lipid suspension (2.5 mL) was added to the vial, followed by 200 µL of FC. Immediately after the addition of the FC, the vial was sealed with a rubber stopper and aluminum crimper cap (Wheaton, Millville, NJ, USA). The vial was then reheated in the hot bath, sonicated at 150 W for 60 s in a bath sonicator (Fisher Scientific) to mix the phases, then shaken at 4400 rpm using an amalgamator (TPC D650 digital amalgamator). Following emulsification, the droplets were quenched in an ice bath and left for 5 min for the emulsion to completely cool. For droplets without GNPs, the solid HC (60 mg) was weighed in a 3 mL glass vial, then heated in a hot bath past its melting temperature. The vial was then quenched in an ice bath, forming an HC film at the bottom of the vial. Lipid suspension (2.5 mL) was then added to the vial, and the remaining fabrication was carried out as described above.

#### 2.1.2 Particle sizing

Number‐percent‐weighted and volume‐percent‐weighted size distributions were measured using a Multisizer 3 Coulter Counter (Beckman Coulter), which sizes particles based on changes in electrical impedance. A 30 µm aperture (size range 0.6–18 µm) was used for all measurements, and three measurements were taken from three separate vials (totaling nine samples) for each droplet formulation.

#### 2.1.3 Fluorescence imaging

Brightfield and fluorescent images were taken on a Nikon Ti‐E widefield microscope equipped with a Hamamatsu Flash 4.0 v3 sCMOS camera. The fluorescent images were taken using a FITC filter (470/525 nm ex/em) with an Excelitas X‐Cite XYLIS LED excitation system. The brightfield and fluorescent images were overlayed using Fiji software.^38^

#### 2.1.4 Thermal vaporization setup

The thermal vaporization temperatures of the exoskeletal droplets with and without GNPs were determined through direct observation under a microscope. Prior to the experiments, the droplets were heat treated in a sealed vial to 5°C above the melting temperature of the HC phase for 5 min, then quenched in an ice bath for additional 5 min. This procedure has been shown to enhance the vaporization efficiency of the endoskeletal droplets.^32^ Following heat treatment, 65 µL of droplets were pipetted into a 15 × 15‐mm incubation well on top of a glass slide. A small k‐type wire thermocouple was placed directly into the sample to monitor the temperature during heating. The microscope slide was then placed on a transparent heating stage controlled by a PID temperature controller (HWPT‐384S Trans Well Plate Heater and mTCII micro–Temperature Controller, Cell MicroControls, Norfolk, VA, USA). The vaporization of the droplets was monitored using an Olympus BX52 microscope and QImaging QIClick CCD Camera. Videos and temperature data of the droplets were recorded as the sample was heated from room temperature to 45°C. The heating rate of the sample followed an exponential rise to the setpoint, which corresponded to a heating rate of approximately ∼0.5°C/s through the vaporization temperature of the sample. Frames of approximately 900 by 670 µm were recorded at 15 frames per second. The activation of the C20 and C21, with and without GNPs, was investigated. For each exoskeletal droplet type, three samples were taken from three different vials (nine samples total). The number of bubbles formed in each video frame was manually counted using the image processing package Fiji and then saved as an array of the number of bubbles per frame. This array was then compared with our recorded temperature per frame data in MATLAB (MathWorks, Natick, MA) to determine the vaporization temperature of each bubble. The data were normalized to the total number of bubbles formed, plotted against temperature, and fitted to a normal cumulative distribution function using Prism 10 (GraphPad, Boston, MA). From the fitted curve, the thermal vaporization temperature (*T_vap_*) was determined, taken as the mean of the normal distribution. To test statistical significance, paired *t*‐tests between the vaporization curve for each droplet subtype were performed in Prism 10.

#### 2.1.5 Megavoltage photon radiation‐induced vaporization setup

Vaporizable exoskeletal droplets incorporating GNPs were engineered to undergo vaporization into ultrasound‐responsive microbubbles upon exposure to MV photon radiation. The design involved leveraging the GNPs to absorb and dissipate radiation, thereby inducing localized heating to vaporize the FC core. Prior to irradiation, the droplets were heated to temperatures between 31 and 34°C, in the vicinity of their thermal vaporization point. The goal was to bring the droplets as close as possible to their thermal vaporization threshold to reduce the amount of irradiation‐induced temperature increase required for activation. As shown in Figure 5b, some vaporization occurred before irradiation. Empirically, we determined that an 8 min heating period was sufficient to ensure that no further droplet vaporization occurred due to heating alone, so that any activation observed after irradiation began would be solely attributed to radiation exposure. Irradiation was performed at a 100 cm distance using a 5 × 5‐cm^2^ 10 MV flattening filter free (10FFF) photon beam from a Varian Truebeam LINAC machine at a dose rate of 24 Gy/min to deliver a total dose of 100 Gy. The dose to the droplet sample is approximated as dose‐to‐water with 0.01Gy/MU at 100 cm source‐to‐surface distance (SSD). The activation behavior of the droplets made with C20, both with and without GNPs, was examined under the MV beam. The experiments involving droplets with GNPs were repeated six times at each temperature, while a single sample of droplets without GNPs was included for comparison, and the resulting vaporization with increasing radiation dose was recorded. Additional control measurements (*n* = 5) were taken by monitoring the activation of droplets with GNPs at the same temperatures in the absence of radiation over the same timeframe (8 min heating period + 4 min, 10 sec additional heating). The microscope data were recorded as videos at 12 frames/sec, synchronized with the entire heating and irradiation process. The gantry angle was tilted to avoid interference with the microscope head, as shown in Figure 1b. The data from the irradiated droplets with GNPs were normalized to the sample's maximum number of bubbles formed, plotted against radiation dose, and fitted to a sigmoidal function. From the fitted curve of droplets with GNPs, the doses necessary to vaporize 10% (D10%) and 50% (D50%) of the droplets at each temperature were estimated. Additionally, the activation threshold at body temperature (37°C) was extrapolated from the fitted data. To compare the extent of vaporization of the control groups to the experimental group (irradiated droplets with GNPs), the control data were normalized to the average of the maximum numbers of vaporized droplets from the experimental group.

#### 2.1.6 Statistical analysis

Two‐way analysis of variables (ANOVA) was performed in Prism 10 to determine the effects of the HC and GNPs on the mean number‐weighted diameter, mean volume‐weighted diameter, and vaporization temperature. Paired *t*‐tests were performed on the vaporization curves to detect statistical differences in the distribution of vaporization temperatures between groups. For the paired t‐tests, a Bonferroni correction factor was employed to account for multiple comparisons (*n* = 3).

To evaluate the statistical significance between experimental (irradiated droplets with GNPs) and control measurements, an unpaired two‐tailed two‐sample t‐test was conducted in MATLAB for each temperature condition, comparing irradiated droplets with GNPs to non‐irradiated droplets with GNPs. A one‐sample *t*‐test was used to compare irradiated droplets with GNPs to irradiated droplets without GNPs at each temperature (the control was treated as the assumed mean). The resulting *p*‐values were reported for both controls: irradiated droplets without GNPs and nonirradiated droplets with GNPs. Statistical analyses were performed in MATLAB, and significance was determined using an alpha of 0.05.

### 2.2 Results

#### 2.2.1 Characteristics of GNP‐doped exoskeletal droplets

**FIGURE 2:**
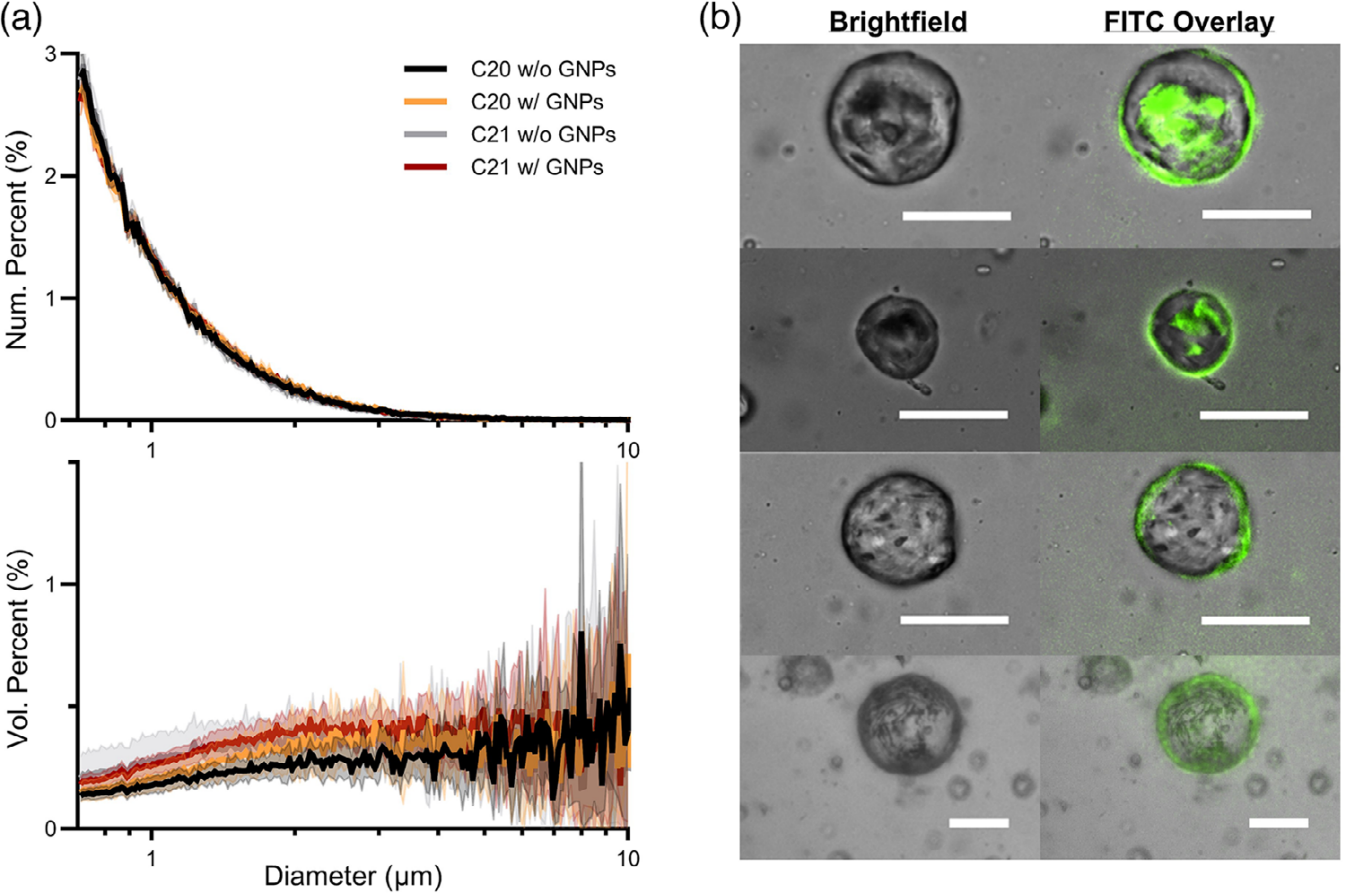
(a) Number and volume‐weighted size distributions of the different exoskeletal droplet formulations. No major differences in the droplet size distributions were observed across the different formulations. (b) Microscopy images of representative larger C20 exoskeletal droplets with FITC‐labeled GNPs. The fluorescent signal overlaps with the exoskeletal droplets, confirming incorporation of the GNPs. The scale bar is 20 µm for all images. GNP, gold nanoparticles.

Size distributions measured for the exoskeletal droplets are shown in Figure 2a. Mean ± standard deviation of the number‐weighted diameters (n = 9 samples) were 1.17 ± 0.02, 1.18 ± 0.01, 1.14 ± 0.04, and 1.16 ± 0.01 µm for the droplets of C20 without GNPs, C20 with GNPs, C21 without GNPs, and C21 with GNPs, respectively. Volume‐weighted diameters were 9.08 ± 1.21, 8.21 ± 2.22, 7.17 ± 2.19, and 6.37 ± 0.76 µm for the droplets of C20 without GNPs, C20 with GNPs, C21 without GNPs, and C21 with GNPs, respectively. Using Two‐Way ANOVA, differences in number‐weighted diameter were significantly different between HC groups (*p* = 0.0056) as well as due to the presence of GNPs (*p* = 0.0278). For volume‐weighted diameters, Two‐Way ANOVA showed a significant difference due to the HC (*p* = 0.0022), but not due to the inclusion of GNPs (*p* = 0.1486). The concentrations were on the order of 1 × 10^9^ droplets per mL for all samples.

Representative images of FITC‐labeled GNP fluorescence overlaid on brightfield images of the droplets are shown in Figure 2b. The brightfield images show the nonspherical morphology expected for the FC‐in‐HC exoskeletal droplets. The fluorescence images confirmed the presence of the GNPs within the droplet and colocalization with the HC phase at the exterior boundaries of the droplets.

#### 2.2.2 Thermal vaporization in the absence of radiation

**FIGURE 3:**
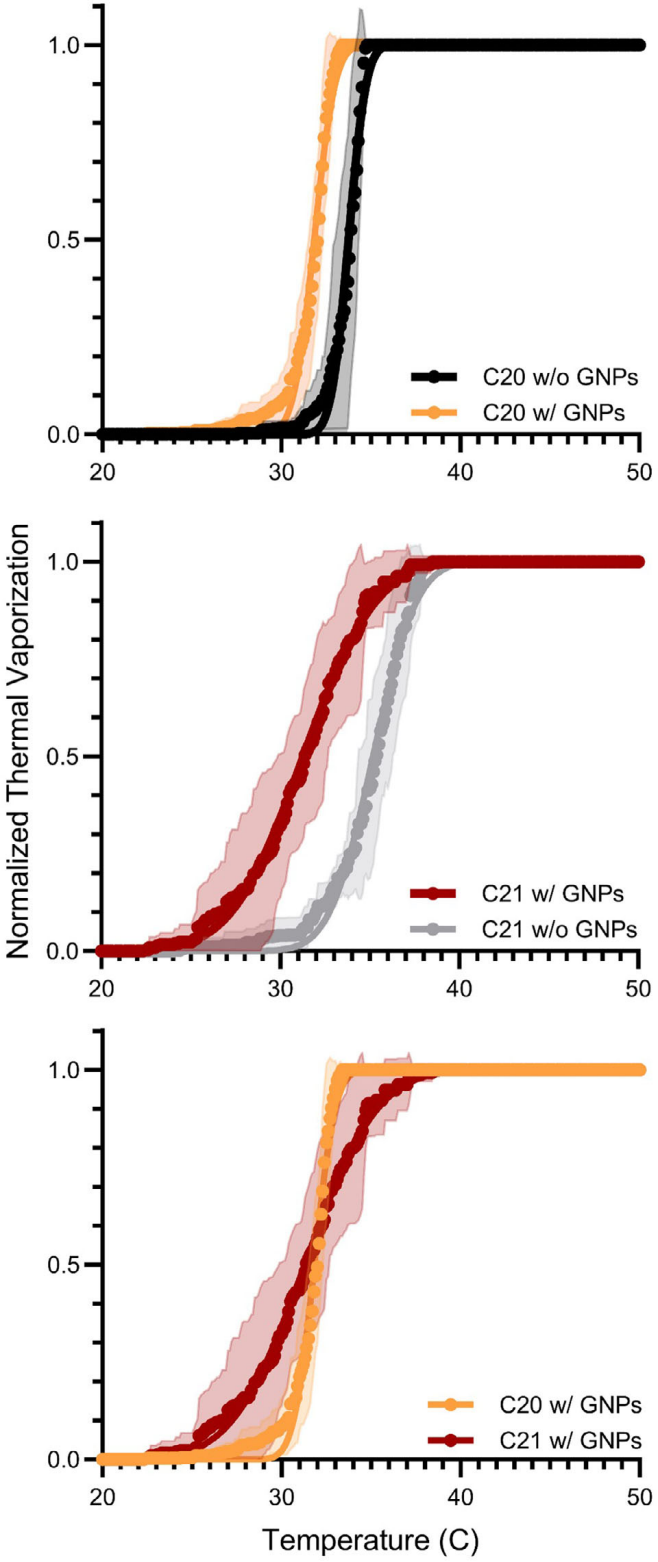
Comparison plots of thermal vaporization curves for C20 w/ GNPs and C20 w/o GNPs, C21 w/ GNPs and C21 w/o GNPs, and C20 w/ GNPs and C21 w/ GNPs. Paired t‐tests showed significant differences for all comparisons. GNP, gold nanoparticles.

**FIGURE 4:**
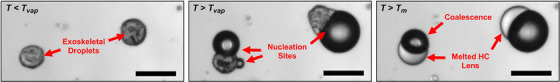
Representative image of two exoskeletal droplets during the heating experiments. As the droplets are heated past their vaporization temperature (T_vap_), bubbles begin to nucleate out of the solid HC. Two separate nucleation sites can be seen on the left droplet. Upon additional heating past the melting temperature (T_m_) of the HC, the HC melts and forms a lens structure on the side of the droplet. The two bubbles from the left droplet coalesce into one bubble. Scale bar is 50 µm. HC, hydrocarbon.

Thermal vaporization curves from the optical heating experiments are shown in Figure 3. The vaporization temperatures (mean ± standard deviation, *n* = 9 samples) were 33.8 ± 0.8, 31.8 ± 0.9, 35.2 ± 1.9, and 31.3 ± 3.2°C for the droplets of C20 without GNPs, C20 with GNPs, C21 without GNPs, and C21 with GNPs, respectively. From Two‐Way ANOVA, the effect of GNPs significantly lowered vaporization temperature (*p* < 0.0001) while the differences due to the HC was not significant (*p = *0.5020). Paired t‐test comparisons of vaporization curves between C20 without versus with GNPs (p<0.0001), C21 without versus with GNPs (p<0.0001) and C20 with GNPs versus C21 with GNPs (p=0.0152) were all significantly different (α=0.0167 after Bonferroni correction). As shown in Figure 4, during heating, the exoskeletal droplets vaporized into bubbles, and subsequent heating melted the HC to form a liquid lens on the bubble surface. Multiple vaporization nucleation sites on a single droplet were sometimes observed to occur. The nucleating bubbles typically coalesced into a single bubble. Representative images of a droplet population prior to and following thermal vaporization can be found in Figure S1 in the Supplementary Material.

#### 2.2.3 Radiation‐induced vaporization

**FIGURE 5:**
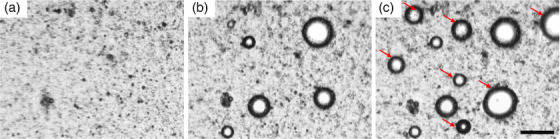
An example of radiation photon‐induced vaporization at 32°C using exoskeletal droplets of C20 with GNPs. (a) Microscopy images of the droplets at room temperature without irradiation, (b) at 32°C before irradiation (thermal vaporization), and (c) after irradiation. Red arrows in (c) show new bubbles formed by vaporization due to the MV beam photon activation. Scale bar is 300µm. GNP, gold nanoparticles; MV, megavoltage.

**FIGURE 6:**
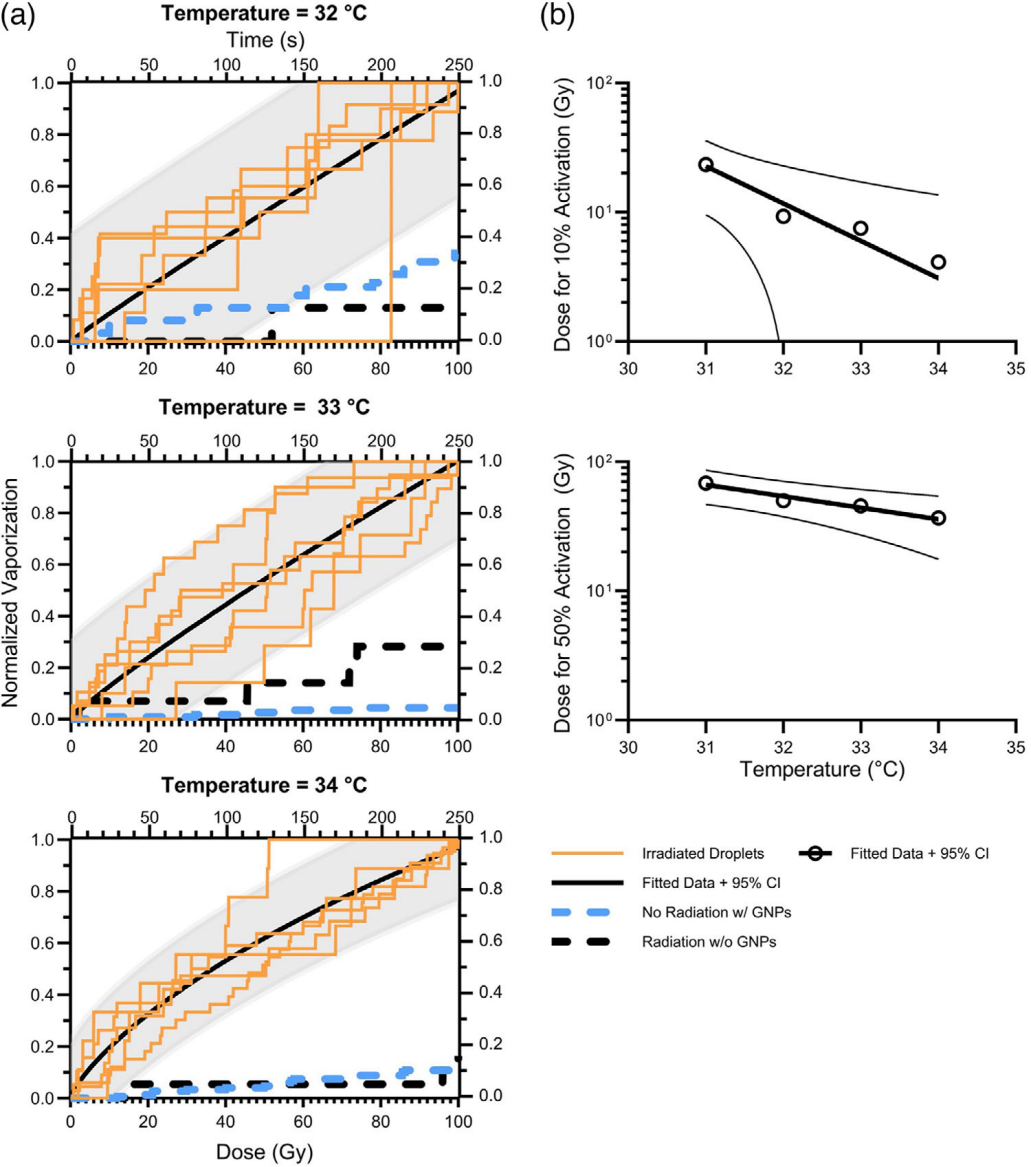
Vaporization of GNP‐doped exoskeletal droplets with an MV photon radiation at different temperatures. (a) Radiation‐induced vaporization at 32, 33, and 34°C. The dose to the droplet sample is approximated as dose‐to‐water with 0.01 Gy/MU at 100 cm SSD. Irradiated exoskeletal droplets without GNPs did not induce significant vaporization, as shown in the black dashed plot. (b) The dose needed to activate 10% and 50% of the resulting radiation‐initiated bubbles. GNP, gold nanoparticles; MV, megavoltage.

Exoskeletal droplets exhibited heightened vaporization upon exposure to MV x‐ray radiation compared to heating alone, as shown in Figures 5 and 6a. Figure 5c depicts the droplets after irradiation, where red arrows indicate the new bubbles formed by vaporization due to MV beam photon activation compared to thermal vaporization before irradiation (Figure 5b). Vaporization of the droplets increased proportionally with escalating radiation dose, while the threshold dose required for vaporization decreased with rising temperature, as shown in Figure 6. Specifically, the dose needed to vaporize 10% of bubbles (D10%) decreased from 22 to 4 Gy, and the dose needed to vaporize 50% of bubbles (D50%) decreased from 67.5 to 36 Gy at temperatures of 31 and 34°C, respectively. After extrapolating the fitted curve in Figure 6b, the activation threshold estimated to occur at body temperature (37°C) was found to be at a clinically relevant dose, with D10% occurring at ∼0.41 Gy. Notably, irradiation of droplets lacking GNPs failed to induce vaporization beyond thermal activation alone. More droplets vaporized by heat alone than by radiation. The ratio of the number of droplets vaporized by heat alone during the 8 min heating period preceding irradiation compared to the number of droplets vaporized by radiation was approximately 2 across all temperatures, as shown in Table 1. The mean ± standard deviation of the number of droplets with GNPs observed to have vaporized ranged from 11.8 ± 5.7 to 40.8 ± 20.6 during the 8 min heating period before irradiation (heat timeframe) and 7.5 ± 3.7 to 18.4 ± 10.0 after irradiation (with radiation timeframe). For control measurements, one experiment was performed at each temperature for droplets without GNPs in the presence of radiation, and five experiments were conducted at each temperature for droplets with GNPs in the absence of radiation. One‐sample *t*‐tests showed statistically more vaporization for the irradiated droplets with GNPs compared to irradiated droplets without GNPs, for all but one temperature and timeframe pair (Table 1). Comparing the nonirradiated droplets with GNPs to the irradiated droplets with GNPs, there was no significant difference in vaporization during the heat timeframe for any of the temperatures prior to radiation. During the radiation timeframe, we observed an increase in vaporization of droplets with GNPs for *T* = 32 to 34°C, compared to similar droplets with GNPs not exposed to radiation (w/o radiation timeframe).

**TABLE 1:** Mean ± standard deviation number of droplets vaporized at each temperature and timeframe. The heat timeframe describes the 8 min incubation period.

Temperature	31°C		32°C		33°C		34°C	
Timeframe	Heat	w/ or w/o Radiation	Heat	w/ or w/o Radiation	Heat	w/ or w/o Radiation	Heat	w/ or w/o Radiation
w/ GNPs w/ Radiation (*n* = 6)	11.8 ± 5.7	7.5 ± 3.7	19.5 ± 19.0	7.7 ± 4.0	31.5 ± 16.8	14.2 ± 4.9	40.8 ± 20.6 (*n* = 5)	18.4 ± 10.0 (*n* = 5)
w/o GNPs w/ Radiation (*n* = 1)	3	1	2	1	12	4	13	3
*p*‐value	0.0127	0.0079	0.0736	0.0094	0.0359	0.0037	0.0392	0.0267
w/ GNPs w/o Radiation (*n* = 5)	21 ± 9.9	3.6 ± 4.2	22.6 ± 4.6	2.6 ± 1.14	46.0 ± 19.1	1 ± 1.4	49.0 ± 16.8	2.4 ± 1.8
*p*‐value	0.0864	0.1378	0.8338	0.0232	0.2128	0.0003	0.5093	0.0080

*Note*: Following this period, droplets were either irradiated (w/ radiation timeframe) or left for an equivalent amount of time on the microscope stage (w/o radiation timeframe). Two sets of controls were performed and compared to the experimental group (irradiated droplets with GNPs): Irradiated droplets without GNPs and non‐irradiated droplets with GNPs. *P*‐values comparing the controls to the associated experimental group are indicated below their mean ± standard deviation counts.

Abbreviation: GNPs, gold nanoparticles.

## 3 DISCUSSION

Here we show for the first time that higher boiling point FC (C_5_F_12_) droplets can be vaporized with MV x‐ray radiation near physiological temperatures when formulated as exoskeletal droplets with GNPs doped into the HC phase. Similar to microbubble ultrasound contrast agents, vaporized droplets are echogenic and can be visualized down to a single bubble per imaging voxel.^21^, ^39^ In the current study, vaporizable exoskeletal droplets were designed with GNPs to increase x‐ray absorption and cause localized dissipation into the HC phase, leading to vaporization. The design involved leveraging the GNPs to absorb and dissipate radiation, thereby inducing localized heating to vaporize the FC core. The resulting microbubbles can be imaged with ultrasound for x‐ray acoustic in vivo dosimetry during RT, with potential for real‐time in vivo dose verification in the application of ART and FLASH‐RT.

In this study, we aimed to leverage the tunable nature of the exoskeletal droplets to achieve photon‐induced vaporization near physiological temperatures. Consistent with previous findings^33^, we observed that increasing the chain length of the HC phase from C20 (*T_m_* = 36°C) to C21 (*T_m_* = 40°C) increased the thermal vaporization temperature of droplets without GNPs (Figure 3). This shift was not seen in formulations containing GNPs; the thermal vaporization temperatures were lower for both formulations upon inclusion of the GNPs. This finding limited the current study to the range of 32 to 34°C: a few degrees below physiological temperature.

This shift toward lower vaporization temperatures may be indicative that the GNPs in the droplets are acting as nucleation sites for vaporization. When solid nucleation sites are present in the liquid FC phase, we expect a reduction in the energy barrier needed for the formation of vapor embryos of sufficient size for vaporization. As a result, vaporization of the liquid will shift away from the spinodal temperature (homogeneous vaporization), toward the bulk boiling temperature of the FC (*T_b_* = 29°C). This hypothesis is further supported by the statistically significant increase in vaporization of the droplets with GNPs during the heat timeframe compared to droplets without GNPs at *T* = 31, 33, and 34°C (Table 1). Previous use of nanoparticles in phase shift emulsions has shown a reduction in acoustic thresholds needed for vaporization during acoustic droplet vaporization (ADV), due to the presence of nucleation sites.^40^, ^41^, ^42^ Vezeridis et al. demonstrated that increasing the concentration of iron‐oxide nanoparticles in perfluorohexane nanodroplets decreased both the thermal vaporization temperature and peak‐negative‐pressure required for acoustic vaporization.^40^ The average (and standard deviation) in loading yield of the GNPs was not measured in this study nor was the effect of GNPs concentrations on the vaporization thresholds; both may play an important role in future optimization of the formulation. Further tuning of the vaporization temperature toward body temperature may be achievable by introducing mixed species of FC in the droplets. Prior studies employing mixed FC systems have shown the ability to tune the thermal^43^ and acoustic thresholds^44^, ^45^ for vaporization.

As an initial proof of concept, a polydisperse droplet size distribution was used for this study (Figure 2). The larger (diameter >30 µm) droplets produced bubbles on the ∼100 µm scale. Such droplets and bubbles are too large for intravenous administration and pose risks of vessel occlusion. We note that common methods to filter and isolate the smaller droplets (e.g., diameter < 1 µm),^46^, ^47^ as well as microfluidic methods to produce droplets of uniform size and morphology,^32^, ^48^ may be used in the future to formulate exoskeletal droplets suitable in size distribution for in vivo use. Additionally, optimization of the HC, FC and GNPs composition and microstructure may be used to increase radiosensitivity. With better radiosensitivity, more energy could be captured from the radiation to overcome the energy barrier for vaporization. This would reduce the need to heat the droplets so close to their vaporization temperature, reducing the number of vaporized droplets prior to radiation exposure (a limitation of this study). Future work will move beyond this proof‐of‐concept study and aim to engineer radiosensitive, monodisperse droplets and employ them as contrast agents for x‐ray acoustic dosimetry for RT therapy. GNPs are generally considered safe for human use, though there are some concerns on the biocompatibility of the HC. We are currently investigating alternative, more biocompatible materials to replace the HC phase with. We have yet to conduct any biocompatibility studies on the formulation.

## 4 CONCLUSIONS

Exoskeletal FC‐in‐HC droplets doped with GNPs demonstrated vaporization upon exposure to MV x‐ray radiation. Considering the echogenic nature of the resultant microbubbles, we propose their potential utilization for real‐time, in vivo x‐ray acoustic dosimetry, alongside other applications.

## CONFLICT OF INTEREST STATEMENT

The authors declare no conflicts of interest.

## Supporting information



Supporting Information
