# Supplementary material for: Radiation induced vaporization of exoskeletal droplets as potential x‐ray acoustic contrast agents
Source: Med Phys. 2025 Aug 8;52(8):e18017. doi: 10.1002/mp.18017 (PMC12334861; doi:10.1002/mp.18017)
Supplement: Supplementary file 1 — Supporting Information [file MP-52-0-s001.doc]

Supplementary Materials for

**Radiation Induced Vaporization of Exoskeletal Droplets as Potential
X-Ray Acoustic Contrast Agents**

**The file includes:**

Sections S1

Table S1

Figure S1

References

**Section S1. Discussion on fugacity of liquid fluorocarbon droplets**

Despite the advantage of higher degrees of superheat, phase-change droplets made with lower boiling point compounds (perfluoropropane, perfluorobutane, and heptafluoropropane) are expected to have a decreased colloidal and *in vivo* stability, owing to their high fugacity at body temperature. Borden et al. showed the main driver of droplet dissolution into a saturated medium is the vapor pressure (
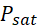
) of the core.1 When the vapor pressure of the droplets is greater than the hydrostatic pressure within the bubble, an Ostwald ripening process of microbubble inflation due to mass transfer from droplets is expected to occur, given by the following expression.


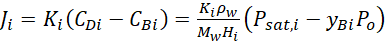
 (1)

where
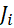
 is the mass flux from the droplets to the bubble (where the index
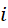
 refers to the fluorocarbon solute),
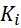
 is the mass transfer coefficient,
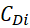
 and
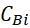
 are the concentrations inside the droplet and bubble, respectively,
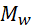
 is the molecular weight of water,
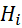
 is Henry’s constant,
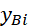
 is the mole fraction in the microbubble,
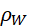
 is the density of water and
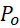
 is the hydrostatic pressure. Comparing to ambient conditions (25 oC,
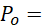
 101.3 kPa
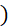
, microbubble inflation is expected to occur for C3F8 (
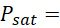
 864 kPa),2 C4F10 (
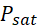
= 266 kPa)2 and C3HF7 (
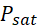
= 453 kPa),2 even for a pure perfluorocarbon microbubble (
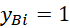
). Bubble inflation is supported by experimental data,3 and is a potential mechanism (in addition to diffusion of dissolved air into the bubble) for the 10x radial expansion of droplets to bubbles observed by Toumia et al.4 (where liquid and vapor molar volumes predict only a 5x expansion), as well as the bubble inflation noted by Carlier et al.5 The surfactant coating the droplets can impact the rate of bubble expansion.3,4 While the polyvinyl-alcohol (PVA) coating may stabilize the droplets to achieve a suitable shelf life due,4 bubble inflation following vaporization still poses a potential safety risk for *in vivo* applications. Higher boiling point FCs, such as perfluoropentane (C5F12, b.p. = 29 °C,
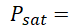
86 kPa
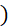
,6 which has a vapor pressure below physiologic pressure (atmospheric + blood pressure,
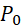
 ~ 130 kPa), is expected to have increased colloidal stability and decreased propensity for bubble inflation.3

|  | **Perfluoropropane**  **(C3F8)** | **Heptafluoropropane**  **(C­3HF7)** | **Perfluorobutane**  **(C­4F10)** | **Perfluoropentane**  **(C5F12­)** | **Perfluorohexane**  **(C6F14)** |
| --- | --- | --- | --- | --- | --- |
| **25** **°C** | 864 kPa | 453 kPa | 266 kPa | 86 kPa | 29 kPa |
| **37 °C** | 1180 kPa | 644 kPa | 384 kPa | 133 kPa | 48 kPa |

**Table S1.** Vapor pressures for fluorocarbons at 25 °C and 37 °C. Vapor pressures for C3F8, C3HF7, C4F10, and C5F12 at 298K and 310K were obtained directly from the U.S. National Institutes of Standards and Technology (NIST).2 Vapor pressure for C6F14 was calculated from the Antoine equation, using fitted values from Stephenson and Malanowski.6

**
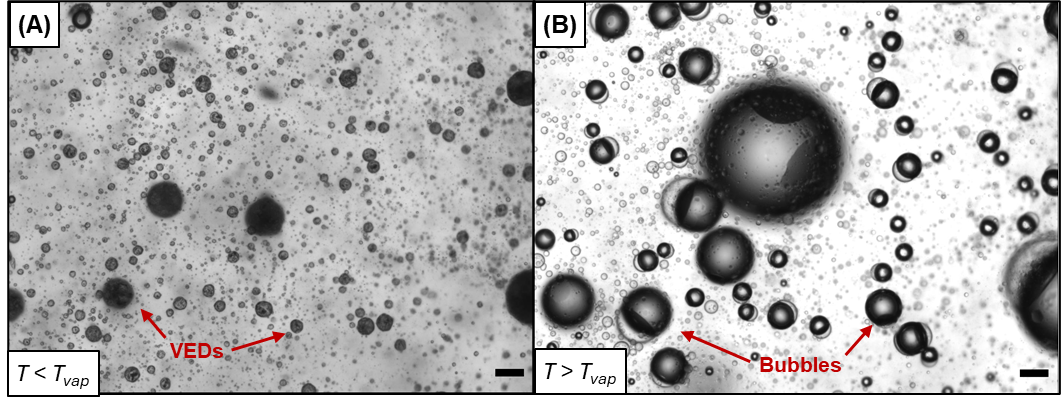
**

**Figure S1.** Micrographs of C20 VEDs w/ GNPs (A) prior to thermal vaporization and (B) following thermal vaporization. VEDs were identified by their opaque, solid structure. Bubbles were identified by their characteristic dark border and translucence. Scale bar is 50 µm.

**References**
